# Outcomes of Severe ARDS COVID-19 Patients Denied for Venovenous ECMO Support: A Prospective Observational Comparative Study

**DOI:** 10.3390/jcm13051493

**Published:** 2024-03-05

**Authors:** Aude Sylvestre, Jean-Marie Forel, Laura Textoris, Ines Gragueb-Chatti, Florence Daviet, Saida Salmi, Mélanie Adda, Antoine Roch, Laurent Papazian, Sami Hraiech, Christophe Guervilly

**Affiliations:** 1 Assistance Publique—Hôpitaux de Marseille, Hôpital Nord, Médecine Intensive Réanimation, 13015 Marseille, France; a.sylvestre@orange.fr (A.S.); jeanmarie.forel@ap-hm.fr (J.-M.F.); laura.textoris@ap-hm.fr (L.T.); ines.gragueb-chatti@ap-hm.fr (I.G.-C.); florence.daviet@ap-hm.fr (F.D.); saida.salmi@ap-hm.fr (S.S.); melanie.adda@ap-hm.fr (M.A.); antoine.roch@ap-hm.fr (A.R.); sami.hraiech@ap-hm.fr (S.H.); 2 Faculté de Médecine, Aix-Marseille Université, Centre d’Études et de Recherches sur les Services de Santé et Qualité de vie EA 3279, 13005 Marseille, France; 3 Centre Hospitalier de Bastia, Service de Réanimation, 604 Chemin de Falconaja, 20600 Bastia, France; laurent.papazian@ap-hm.fr; 4 Unité des Virus Émergents (UVE: Aix-Marseille Univ, Università di Corsica, IRD 190, Inserm 1207, IRBA), 13284 Marseille, France

**Keywords:** venovenous extracorporeal membrane oxygenation, acute respiratory distress syndrome, COVID-19, contraindication, outcome, recovery

## Abstract

**Background**: Few data are available concerning the outcome of patients denied venovenous extracorporeal membrane oxygenation (VV-ECMO) relative to severe acute respiratory distress syndrome (ARDS) due to COVID-19. **Methods**: We compared the 90-day survival rate of consecutive adult patients for whom our center was contacted to discuss VV-ECMO indication. Three groups of patients were created: patients for whom VV-ECMO was immediately indicated (ECMO-indicated group), patients for whom VV-ECMO was not indicated at the time of the call (ECMO-not-indicated group), and patients for whom ECMO was definitely contraindicated (ECMO-contraindicated group). **Results**: In total, 104 patients were referred for VV-ECMO support due to severe COVID-19 ARDS. Among them, 32 patients had immediate VV-ECMO implantation, 28 patients had no VV-ECMO indication, but 1 was assisted thereafter, and 44 patients were denied VV-ECMO for contraindication. Among the 44 patients denied, 30 were denied for advanced age, 24 for excessive prior duration of mechanical ventilation, and 16 for SOFA score >8. The 90-day survival rate was similar for the ECMO-indicated group and the ECMO-not-indicated group at 62.1 and 61.9%, respectively, whereas it was significantly lower (20.5%) for the ECMO-contraindicated group. **Conclusions**: Despite a low survival rate, 50% of patients were at home 3 months after being denied for VV-ECMO for severe ARDS due to COVID-19.

## 1. Introduction

The use of venovenous extracorporeal membrane oxygenation (VV-ECMO) support for severe and refractory acute respiratory disease syndrome (ARDS) patients has dramatically increased worldwide, especially since the coronavirus disease 2019 (COVID-19) pandemic, with 17,509 VV-ECMO runs recorded in the ELSO Registry [1]. A recently published high-quality meta-analysis [2] involving 42 observational studies encompassing 17,449 patients supported by VV-ECMO for COVID-19 severe ARDS reported that the main factors associated with mortality were older age; male sex; chronic lung disease; and pre-cannulation factors, such as the longer duration of symptoms, longer duration of invasive mechanical ventilation, higher partial pressure of arterial carbon dioxide, and higher driving pressure.

Even if the clinical efficacy of VV-ECMO support in severe ARDS is recognized [3,4], VV-ECMO is a highly invasive procedure with potentially life-threatening complications, such as major bleeding, venous thromboembolism events [5], or cannula infections [6]. In addition, a significant number of ECMO survivors can have severe disabilities one year after ICU admission [7].

Moreover, VV-ECMO support comes at subsequent costs, and resources are more constrained during a pandemic. In addition, COVID-19 patients have longer VV-ECMO run durations than other etiologies [8], and the accuracy for outcome prediction using clinical severity scores (RESP and PRESERVE scores) validated for non-COVID-19 patients seems relatively weak for COVID-19 patients [9,10].

Little is known regarding the prognosis of patients for whom VV-ECMO was contraindicated or not indicated. We hypothesize a very high hospital mortality (e.g., >80%) for patients for whom ECMO was contraindicated and a lower mortality rate for patients not indicated for ECMO as compared with those who actually received ECMO.

Therefore, the main objective of the study was to evaluate the outcome of patients denied VV-ECMO support. The 90-day survival was the main outcome of comparisons between groups.

## 2. Methods

### 2.1. Study Design and Setting

This prospective observational comparative cohort study was conducted in the medical ICU of the North University Hospital in Marseille, France, which is a regional VV-ECMO referral center for acute respiratory failure. The study received institutional approval (n°2020-53). The need for individual informed consent was waived for this retrospective analysis of data collected prospectively for routine care, with no breach of privacy or anonymity.

The service manages 50 to 60 VV-ECMO patients annually, with a 24 h VV-ECMO hotline attended by a senior ICU physician for VV-ECMO referral. All patients for whom the H24 hotline was consulted for a potential indication of VV-ECMO between November 2020 and June 2021 were included in this study. Our center is the regional referral VV-ECMO center of an area with 5 million inhabitants.

### 2.2. Decision-Making Process, ECMO Criteria, and Initial ECMO Management

The 24 h VV-ECMO hotline is monitored by an attending ICU senior intensivist.

Baseline patient’s characteristics, comorbidities, the etiology of ARDS, the duration of mechanical ventilation, mechanical ventilation parameters, blood gases, adjunctive ARDS therapy, and SOFA score were recorded during the first telephone contact time point and kept in a database. Patients considered for VV-ECMO by our regional VV-ECMO center had 3 possible trajectories: 1/VV-ECMO support was indicated, and the patient was retrieved by the mobile VV-ECMO team; 2/VV-ECMO was not indicated at the time of the call because the patient did not meet the severity criteria for VV-ECMO support but needed re-evaluation in the case of deterioration; 3/patient was definitely contraindicated relative to VV-ECMO. The decision-making process to contraindicate ECMO required a consensus agreement from 3 senior intensivists.

The criteria for VV-ECMO support were those of the EOLIA trial [4]. The criteria are as follows: a PaO_2_:FiO_2_ ratio of less than 50 mm Hg for more than 3 h; a PaO_2_:FiO_2_ of less than 80 mm Hg for more than 6 h; or an arterial blood pH of less than 7.25 with a PaCO_2_ of at least 60 mm Hg for more than 6 h.

The reasons to decline a patient were as follows: advanced age over 65 years, severe comorbidities (e.g., active lung cancer), multiple organ failure assessed by the Sequential Organ Failure Assessment (SOFA) score, and duration of mechanical ventilation or contraindication to anticoagulation. The SOFA score is a multi-organ failure scoring system including the cardiovascular system, respiratory function, central nervous system, renal function, liver function, and coagulation [11]. Each organ failure is scored from 0 to 4. Global scores range from 0 (no organ failure) to 20. For example, severe ARDS with a PaO_2_:FiO_2_ ratio of less than 100 mm Hg is scored 4 points.

Patients who did not meet VV-ECMO criteria either remained at the referring center with advice for respiratory support or were transferred to the expert center on conventional mechanical ventilation by the mobile emergency and resuscitation services team if PaO_2_:FiO_2_ was at least 100 mmHg and they exhibited hemodynamic stability. For patients for whom VV-ECMO was decided, a modified mobile team was implemented, including a senior cardiothoracic surgeon and a senior intensivist, and an ECMO perfusionist was dispatched to the referring center. In this case, the patient was cannulated at the bedside and transferred to the expert center on VV-ECMO support.

Technical aspects and ECMO management have been previously described [12]. Briefly, we first utilized the right femoral–right jugular vein configuration using an ultrasound-guided and percutaneous Seldinger approach, with large bore cannulas of 27–29 F in order to allow the inflow in achieving an ECMO blood flow of 4–6 L per minute; centrifugal pumps (Bio-console 560; Medtronic Perfusion Systems, Minneapolis, MN, USA), prolonged heparin-coated tubing, and Quadrox D oxygenators (Getinge, Maquet, Goteborg, Sweden) were used to achieve arterial blood O2 saturation of 90–95%, while the oxygen fraction delivered by the oxygenator was set at 1. The sweep gas flow of oxygen was progressively increased to achieve a pH value of >7.3 and PaCO_2_ of <45 mmHg. Before the transfer, abdominal and chest X-rays were performed to ensure the adequate positioning of the tips of the cannula with sufficient distance to avoid important recirculation. After canulation, during transport, the ventilator was set in volume-controlled mode with tidal volume of 2–4 mL/kg of predicted body weight, the respiratory rate was set between 10 and 15 cycles per minute, and we settled a PEEP ≥ 10 cmH_2_O to achieve plateau pressure ≤ 25 cmH_2_O and driving pressure ≤ 15 cmH_2_O.

### 2.3. Data Collection

The following data were extracted from the call log database of the VV-ECMO hotline, the patient’s computed file if the patient was transferred to our center, or the medical file of the referral initial ICU:

Demographic characteristics and severity score at ICU admission;

Duration of mechanical ventilation, ICU and hospital length of stay, mortality at D60 and D90, and the number of instances of withdrawn life-sustaining therapy;

Each referring center was contacted to obtain data during hospitalization. If patients were discharged before day 90, they were contacted by phone to investigate their status after hospital discharge.

### 2.4. Statistical Analysis

Descriptive statistics included the number and percentages for categorical variables and medians and interquartile ranges (IQRs) for continuous variables. Comparisons were performed with the Kruskal–Wallis test for continuous variables and the Chi^2^ test for categorical variables. We did not use multiple imputations for missing data. Kaplan–Meier survival curves were compared using the log-rank test. A two-tailed *p* ≤ 0.05 was considered statistically significant. Statistics and figures were performed with SPSS 20.0 (SPSS Inc., Chicago, IL, USA).

## 3. Results

**Figure 1:**
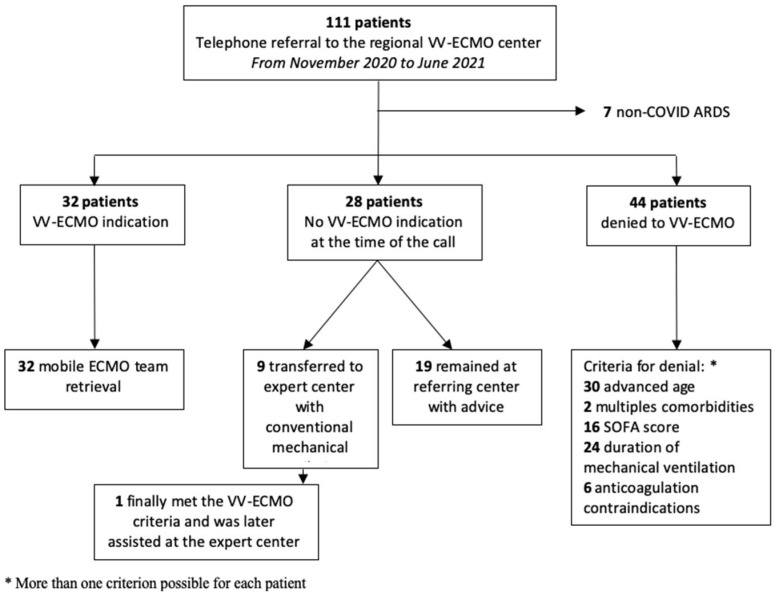
Flowchart.

From November 2020 to June 2021 (third COVID-19 wave in the southeast of France), 111 patients were considered for ECMO-VV support by the regional VV-ECMO center via the VV-ECMO hotline. Figure 1 shows the study’s flowchart, and Table 1 shows the patient’s main characteristics. Among these 111 patients, 104 were COVID-19 patients. Thirty-two patients exhibited a VV-ECMO support indication and were retrieved on VV-ECMO by our mobile team. Twenty-eight patients had no VV-ECMO indication criteria at the time of the call. Among them, 19 remained in the calling center due to management advice, and 9 were transferred to the regional VV-ECMO center with conventional mechanical ventilation. One of the transferred patients later met the VV-ECMO support criteria and was assisted after the transfer. In total, 44 patients were declined due to one or more VV-ECMO contraindications: 30 patients (68%) were declined due to an advanced age of over 65 years, 24 (55%) were declined due to having a mechanical ventilation duration of >7 days, 16 (36%) were declined for multiple organ failure, 6 (14%) had a contraindication for anticoagulation, and 2 (5%) were denied because of multiple comorbidities (Figure 1).

### 3.1. Baseline Characteristics

Baseline characteristics of patients on the day of the call were comparable in terms of comorbidities and body mass index, but denied VV-ECMO patients were older (*p* < 0.001) and experienced longer mechanical ventilation durations before the call (*p* = 0.002), which is consistent with our selection criteria. The SOFA score on the day of the call was lower in the no-VV-ECMO-indication group. In the group of patients denied VV-ECMO, 36% were contraindicated for high SOFA > 8. Besides 4 points due to respiratory failure, those patients had septic shock and acute renal failure associated. At the time of the call, patients indicated and contraindicated for VV-ECMO had lower PaO_2_:FiO_2_ and lower pH due to PaCO_2_ as compared with the patients not indicated for VV-ECMO. Respiratory mechanics parameters were comparable between the three groups, and patients were all managed according to a lung-protective ventilation strategy, with tidal volumes around 6 mL/kg of the predicted body weight and a moderate to high PEEP (median of 12 with an IQR of 10–14 cm H_2_O). All patients were receiving continuous neuromuscular blocker infusion. Regarding prone positioning, 97% of patients in the VV-ECMO-indicated group, 100% of patients in the VV-ECMO-not-indicated group, and 98% of patients in the VV-ECMO-contraindicated group received at least one prone position session. Plateau pressure and driving pressure were comparable between the groups. Concerning gas exchanges, patients who were not indicated relative to VV-ECMO had a higher P/F ratio and pH and lower PaCO_2_ at the time of the call compared to the other groups. Patients of the VV-ECMO-indicated group and the VV-ECMO-contraindicated group had similar arterial blood gas consistent with our selection criteria (Table 1).

### 3.2. Outcomes and Follow-Up

**Figure 2:**
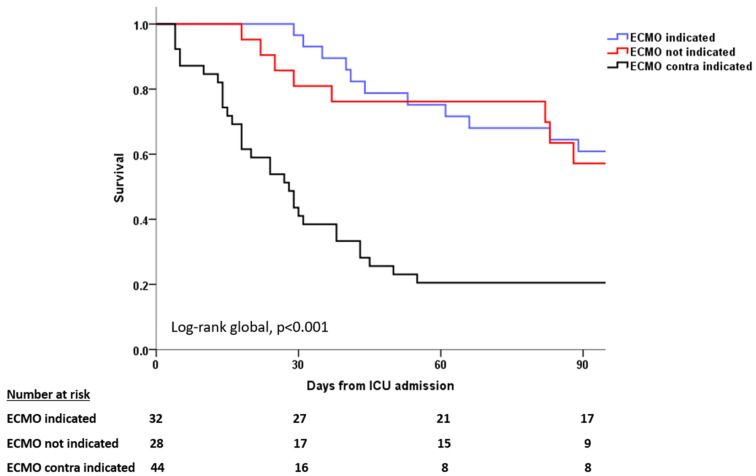
Kaplan–Meier survival analysis at 90 days.

The day-90 survival status was available for 29 (90.6%) of the patients retrieved on VV-ECMO, 21 (75%) of the patients with no VV-ECMO indication, and 39 (88.6%) of the declined patients. For the patients in whom day-90 survival was available, 21 (62.1%) patients retrieved on VV-ECMO, 20 (61.9%) patients with no VV-ECMO indication, and 13 (20.5%) declined patients survived on day 90 (*p* < 0.001 via log-rank test) (Table 2 and Figure 2). Patients not indicated for VV-ECMO had a nonsignificant lower number of instances of withdrawn life-sustaining therapy as compared with the patients indicated and those contraindicated for VV-ECMO.

Patients of the VV-ECMO-indicated group had a significantly longer median duration of mechanical ventilation at 39 (25–53) days compared to 20 (12–46) days in the VV-ECMO-not-indicated group and 23 (8–39) days in the VV-ECMO-contraindicated group (*p* = 0.002). Patients of the VV-ECMO-indicated group also had significantly longer ICU and hospital stay compared to the VV-ECMO-not-indicated group and the VV-ECMO-contraindicated group, with a median of 53 (31–66) days versus 29 (22–61) days and 29 (14–46) days (*p* = 0.001) and 66 (38–83) days versus 36 (24–59) days and 29 (15–49) days (*p* < 0.001), respectively (Table 2).

At the last follow-up, among the patients who survived, 14 (78%) of the VV-ECMO-indicated patients, 9 (56%) of the VV-ECMO-not-indicated patients, and 6 (50%) of the VV-ECMO-contraindicated patients returned home. Four (22%) of the VV-ECMO-indicated patients, three (18%) of the VV-ECMO-not-indicated patients, and six (50%) of the VV-ECMO-contraindicated patients were in the rehabilitation center. Compared to four (25%) of the VV-ECMO-not-indicated patients, none of the VV-ECMO-indicated and contraindicated patients were still in the ICU or a mechanical ventilation weaning unit (Table 2).

## 4. Discussion

Our study reports the outcomes of severe ARDS COVID-19 patients referred to our regional center for VV-ECMO during the third COVID-19 wave in France. If the 90-day survival rate was comparable between the patients who received VV-ECMO support and those who did not meet the severity indication criteria and were managed on conventional mechanical ventilation, patients denied for VV-ECMO had a very low 90-day survival. A similar 90-day survival rate was observed in the ECMO-indicated patients and the ECMO-not-indicated patients, which is reassuring and seems to validate a posteriori the restrictive ECMO selection criteria that we defined for patients with COVID-19. However, we cannot exclude that some patients in the ECMO-not-indicated group may have received ECMO for another etiology of ARDS, and that it may have improved their outcomes.

Interestingly, in our cohort, 20.5% of patients presenting the VV-ECMO support criteria but contraindicated because of advanced age, long duration of mechanical ventilation, multiple comorbidities, or multiple organ failure survived at day 90 after ICU admission. There are very few data in the literature regarding the outcomes of patients denied for VV-ECMO support. One UK study conducted in severe ARDS patients before the COVID-19 era reported 16.6% with respect to 6-month survival in patients denied VV-ECMO support [13]. During the first wave of COVID-19 in Greater Paris, Levy et al. reported a 90-day survival of 14% in patients denied for VV-ECMO [14].

For patients indicated for VV-ECMO, the 90-day survival rate of 62.1% of our cohort is consistent with large studies and registries [15,16], and it is slightly higher than the rate reported in a recent randomized controlled study assessing the continuation of the prone position during VV-ECMO [17].

The COVID-19 variant, especially delta, could have a negative prognostic impact on COVID-19 patients supported by VV-ECMO [16]. In the present study, the inclusion period encompasses the predominance of alpha and delta variants in France.

Ninety-day survival was comparable in patients retrieved on VV-ECMO support and patients left on conventional mechanical ventilation because they did not meet the VV-ECMO support criteria.

These results comfort us in our selection method for VV-ECMO support in severe ARDS COVID-19 patients. Several scores have been proposed to help the physician in this difficult decision-making process, such as the PRESERVE mortality risk score [18], the RESP score [19], or the ECMOnet score [20]. However, their accuracy in predicting COVID-19 patient mortality under VV-ECMO seems uncertain [9,21]. Gannon et al. have proposed a specific classification and regression tree to predict in-hospital mortality for COVID-19 patients eligible for ECMO [10].

We also agree that our results may not be generalized to other cases of severe ARDS. However, in a large UK study that found a very close survival rate for patients denied VV-ECMO [13], only 10% of patients had viral pneumonia as the leading cause of ARDS.

In our cohort, advanced age was the main reason for the denial of VV-ECMO support because strong evidence in the literature shows an increased mortality in patients aged 65 or above [22,23], notably in countries such as Germany where there were few resource restrictions even during the COVID-19 pandemic that influenced and resulted in a liberal indication for ECMO, and 90-day mortality as high as 80% for patients aged 60 years or older [24]. The duration of invasive mechanical ventilation was the second main reason for denial relative to VV-ECMO. A period of mechanical ventilation greater than 7 days has been proposed as a condition for declining VV-ECMO for severe ARDS COVID-19 patients [25]. However, this was not confirmed as a prognostic factor in an Austrian retrospective study [26].

A high SOFA score was not observed to be a determinant selection criterion in our cohort, with a comparable mean SOFA score in the VV-ECMO-indicated and VV-ECMO-contraindicated groups. This might be explained by the first selection in the ICU calling centers that do not propose VV-ECMO as a support for patients with multiple organ failure. In addition, we did not have to face triage situations in the south of France; thus, none of the patients in our cohort were denied because of a lack of ICU bed availability or VV-ECMO machines. However, the specific context of the global COVID-19 pandemic may have impacted our results.

This prospective, monocenter study has some limitations beyond its small sample size. First, some data were missing due to the difficulty in retrieving data from the referring center and the long-term follow-up for non-indicated patients or patients denied VV-ECMO. A larger multicenter study might help confirm our report. Second, our study was not designed to assess functional recovery in survivors, which is of substantial interest. Interestingly, a recent retrospective multicenter study observed no significant differences in the markers of pulmonary, neurocognitive, or psychiatric functional recovery outcomes when comparing a contemporaneous clinic-based cohort of ARDS survivors who were managed with ECMO to those without ECMO [27].

## 5. Conclusions

In this prospective observational comparative study, the main reasons to deny VV-ECMO were advanced age, excessive prior duration of mechanical ventilation, and high SOFA score. Unexpectedly, almost 20% of COVID-19 patients denied VV-ECMO were alive 3 months after ICU admission. Larger studies are needed to improve the prediction accuracy of VV-ECMO success, especially in COVID-19 etiology.

## Figures and Tables

**Table 1:** Baseline characteristics of patients considered for VV-ECMO support at the time of the call.

	VV-ECMO Indicated (n = 32)	VV-ECMO Not Indicated (n = 28)	VV-ECMO Contra-Indicated (n = 44)	*p*
Age, years	59 (55–62)	53 (47–60)	66 (60–69)	<0.001
Male	29 (90)	19 (68)	36 (82)	0.08
Body mass index, kg/m^2^	30.9 (25.7–33.7)	30.1 (27.1–35.0)	28.7 (26.1–36.7)	0.97
Comorbidities				
Hypertension	13 (40)	8 (28)	19 (43)	0.44
Diabetes	9 (28)	7 (25)	19 (43)	0.20
Chronic lung disease	8 (25)	3 (11)	9 (20)	0.36
Cardiomyopathy	4 (12)	4 (14)	9(20)	0.61
Immunocompromised	4 (12)	1 (4)	3 (7)	0.41
SOFA score at call	6 (4–8)	4 (4–7)	7 (4–8)	0.01
Duration of mechanical ventilation prior call, days	4.5 (1–7)	2.5 (1–7)	7 (3–17)	0.002
Respiratory parameters at time of call				
FiO_2_, %	100 (100–100)	80 (70–100)	100 (85–100)	<0.001
PEEP, cmH_2_O	12 (10–15)	12 (10–14)	10 (9–14)	0.17
Tidal volume, mL/kg/PBW	6.2 (5.5–6.8)	6.1 (5.8–6.7)	6.1 (5.8–6.7)	0.68
Respiratory rate, cycles/min	30 (24–32)	28 (22–29)	30 (26–32)	0.01
Plateau pressure, cmH_2_O	32 (27–35)	30 (25–32)	30 (28–31)	0.25
Driving pressure, cmH_2_O	17 (13–24)	16 (13–21)	19 (15–22)	0.32
RS static compliance, mL/cmH_2_O	25 (17–28)	25 (17–32)	20 (16–25)	0.09
Arterial blood gas at time of call				
pH	7.29 (7.25–7.36)	7.38 (7.33–7.45)	7.31 (7.25–7.39)	0.001
PaO_2_:FiO_2_, mmHg	71 (56–84)	100 (75–130)	70 (61–91)	0.004
PaCO_2_, mmHg	60 (56–72)	53 (45–60)	63 (56–67)	0.001
Lactates, mmol/L	1.5 (1–2.75)	1.4 (1.1–1.7)	1.5 (1–2)	0.84
Concomitant or prior treatments at time of call				
Continuous NMBA infusion	32(100)	28 (100)	44 (100)	-
Prone positioning	31 (97)	28 (100)	43 (98)	0.66
Number of prone positioning sessions	2 (1–4)	1 (1–3)	3 (1–4)	0.15
Inhaled nitric oxide	21 (65)	14 (50)	28 (64)	0.36
Almitrine infusion	3 (9)	0 (0)	5 (11)	0.18
Vasopressor infusion	15 (47)	10 (37)	20 (45)	0.63
Renal replacement therapy	2 (6)	1 (3)	4 (9)	0.65

Data are expressed as a median and interquartile range or a number and %. Comparisons were performed using the Kruskal–Wallis test for continuous variables and the Chi^2^ test for categorical variables. Abbreviations: VV-ECMO, venovenous extracorporeal membrane oxygenation; SOFA, sequential organ failure assessment; PEEP, positive end-expiratory pressure; RS, respiratory system; NMBA, neuromuscular blocking agents.

**Table 2:** Outcomes of patients according to the VV-ECMO support decision.

	VV-ECMO Indicated	VV-ECMO Not Indicated	VV-ECMO Contra–Indicated	*p*
Death by day 60, n (%) n = 94	10 (32.3)	7 (31.8)	31 (75.6)	<0.001
Lost of follow up by day 60, n (%) n = 10	1 (3.1)	6 (21.4)	3 (6.8)	0.04
Death by day 90, n (%) n = 89	11 (37.9)	8 (38.1)	31 (79.5)	<0.001
Lost of follow up by day 90, n (%) n = 15	3 (9.4)	7 (25)	5 (11.4)	0.17
Withdraw life-sustaining therapy, n (%) n = 102	10 (31.2)	2 (7.4)	13 (30.2)	0.055
Duration of mechanical ventilation, days n = 99	39 (25–53)	20 (12–46)	23 (8–39)	0.002
Duration of ICU stay, days n = 100	53 (31–66)	29 (22–61)	29 (14–46)	0.001
Duration of hospital stays, days n = 93	66 (38–83)	36 (24–59)	29 (15–49)	<0.001
Last follow up n = 46				0.023
At home	14 (78)	9 (56)	6 (50)	
Rehabilitation center	4 (22)	3 (18)	6 (50)	
Still in ICU/Mechanical ventilation weaning unit	0 (0)	4 (25)	0 (0)	

Data are expressed as a median and interquartile range or a number and %. Comparisons were performed using the Kruskal–Wallis test for continuous variables and the Chi^2^ test for categorical variables. VV-ECMO, venovenous extracorporeal membrane oxygenation; ICU, intensive care unit.

## Data Availability

The datasets used and analyzed during the current study are available from the corresponding author upon reasonable request.

## Abbreviations

ARDS	acute respiratory distress syndrome
COVID-19	coronavirus disease 2019
ELSO	extracorporeal life support organization
ICU	intensive care unit
IQR	inter quartile range
NMBA	neuromuscular blocking agents
PEEP	positive end-expiratory pressure
P/F ratio	PaO_2_ to FiO_2_ ratio
RS	respiratory system
SOFA score	sequential organ failure assessment score
VV-ECMO	venovenous extracorporeal membrane oxygenation
